# Looking Ahead to the Future...

**DOI:** 10.21470/1678-9741-2018-0601

**Published:** 2018

**Authors:** Rui M. S. Almeida

**Affiliations:** 1 President of Brazilian Society of Cardiovascular Surgery (SBCCV)

***"We are made wise not by the recollection of our past, but by the responsibility for our future."***

**George Bernard Shaw**

A new year begins and, with it, great new challenges come along. At a time when we see our specialty with important issues, we have to focus on what can be done in the near future. This look must always be based on all the achievements made in previous years. We remind you that the changes in our Medical Residency, started more than 15 years ago by Prof. Gilberto Barbosa with an arduous effort of some selfless colleagues, coming to its approval by the final work of Profs. Fabio Jatene and Henrique Murad. We can not forget the tireless struggle to improve the conditions of treatment of patients with congenital heart disease and all those involved in this difficult task of performing pediatric cardiac surgeries, which have been declining annually in numbers, culminating in the approval of readjustments in the Unified Health System (SUS) payments. Thus, there will be an increase in the number of procedures to be performed and a large number of pediatric patients will benefit.

All these achievements need to be settled, which will be a great challenge. However, the two major ideals of a group of surgeons - Medical Education and Professional Defense - begin to become reality.

In Medical Education we have seen the approval, by the National Medical Residency Commission (CNRM), of our Medical Residency with direct access, without the need to perform General Surgery, and with that the consequent reduction for five years of its period. But there is much to be done. We need to re-evaluate the Training Centers (TC), and know which ones are apt to receive and adequately prepare our future cardiovascular surgeons; to adapt the unification, in all TCs, of the annual evaluation of progression of the residents, based on competences and a careful evaluation to be carried out simultaneously in all of them, being this responsibility of the Brazilian Society of Cardiovascular Surgery (SBCCV); it will also be necessary to define the annual competences for each cycle of the Medical Residency. All these implementations demonstrate the firm purpose of our Society in constantly improving the training of our professionals.

The initial impetus for a greater demand for our specialty is based not only on this change of Residency, but also on the continuity of the support given to our Academic Leagues and, consequently, the increase in demand for the specialty. Support for Academic Leagues has been an important work developed by the SBCCV for 6 years and we have already reaped the fruits. We have developed partnerships and some very successful work fronts, such as the blog of the Brazilian Journal of Cardiovascular Surgery (BJCVS), administered by a team of engaged and committed academics. Thus, in addition to allowing contact with cardiovascular surgeons, we promote scientific initiation with the encouragement of writing articles. The idea is to identify leaders, potential reviewers and thus shape the future of the SBCCV.

Another important point is Professional Defense. It is unacceptable that our patients do not have access to the new technologies or guarantee that the surgeries will be performed in the most appropriate and safe way. The whole environment in which these procedures are performed must have the ideal conditions, not the minimum ones, to optimize the quality of care and patient survival. In the near future, we will continue to seek solutions so that our patients can have the best form to treat their cardiovascular diseases, always based on the ethical and moral principles of our medical oath and professional codes of conduct and of our Society.

We are aware of the fact that there are a myriad of problems that are occurring day by day and for which the solution, unlike in the operative field, is often not ours but, without a doubt, with the collective work we will reach the solution. Our union will always be the asset, so that we can achieve great victories and continue to carry out what we have all proposed from the beginning - to treat our patients with the quality they need and to have at their disposal all the professional expertise we have.

The path is long and tortuous, both for the problems we are facing and for the diversities of this great Brazil. But we all have in common the will to change and, above all, our workforce.

We need to adapt to the changes taking place around us and to project our future. For this, we have to adapt the SBCCV to the new times, correcting directions to make it agile and suitable to all its associates. We need, looking back and always keeping in mind the history of our society and what has been achieved until now, reinventing the SBCCV, leaving it ready for the new challenges of the 21^st^ century. Undoubtedly, for this purpose, the words of each of the cardiovascular surgeons are important, and we will achieve this through unity and joint work, based on assistance, academicism, research and, above all, on associativism, from which our strength derives, make our specialty one of the most important in the national and international scenarios. The responsibility for the future belongs to us and therefore we will all, from now on, continue the work for a strong, dynamic and modern SBCCV.

